# Supplementary material for: Chromothripsis during telomere crisis is independent of NHEJ, and consistent with a replicative origin
Source: Genome Res. 2019 May;29(5):737–49. doi: 10.1101/gr.240705.118 (PMC6499312; doi:10.1101/gr.240705.118)
Supplement: Supplemental Material [file supp_gr.240705.118_Supplemental_file_1.zip › contigs/annotated_contigs/DB76/contig.4.DB76_length_1270_mean_cov_18.5220472441.docx]

**DB76_length_1270_mean_cov_18.5220472441**

```
CATATGATTCAAATTTAGTAACATATTACATGTTATAAACTCAAATTTGCACTTAACGAACAAAAATATTACACTTCACTGCTGTTCAT
 >chr9:25184551-25184899 - E=3e-197
ACTCTCACTGCTTTACCTTTTTTTATTGTGGTAAAAAACATATAACATAAAATTTACCATCTTATTTCTAAATGTACAATTGAGTAGTG

TTAAGCATGTTCACATGGTTGTGCAACCAATCTTCAGAACTTTTCATCTTGCAAAACTGAAACTCTACATATTAAGTGATAGCTCCGCT

TTTCTCCTTCCTCCCAGCCCCTGGCAACTACCATTATGCTTTCTATTTCTATGAGTTTGATTACACAAGATACCTCATATC|T|TGGGA
                                                                                   >chr
GAAAATTTTCACAACCTACTCATCTGACAAAGGGCTAATATCCAGAATCTACAATGAACTCAAAGAAATTTACAAGAAAGAGCAGTTTT
11:49486046-49486436 + E=2e-219
AAGAAGGATGTTTACAACAATAAATGCCTACATTAACAAAAAAAATTCCAAATACATAATCCAATGCTGTACTTCAAAAAACTGAAAAT

AAAGGTCAAACTATGCCTAAAGTTAACAGAAGGAAGGAAATAATAAAGATCAGAGAAGAAGTAAATGAAAATAGCACAAAAGATCAATG

AAGCTAAGAATTGGTTTTTAACAAGGTAAACAAAATTGACATACCTTTATCGAGAATAAGAAAAAAAAAGACGCAAATAAATACAATCA

GAAATGAAAGAGGAGACATTACAACAG|AT|TCCTGTAAAAGCCATTTGGATGACCCAATGGGGTGAGTTTACAGGGAAGCGCTCCCTG
                            >chr20:49266242-49266445 + E=5e-110
AGGGCTAGGCTGGCTGGAAGCAGATACTTTGCCCCCTTCCAAGGACGGTAATGAGGTTTGAGGCTTCCAATGGGGGCAGGGGATGATCT

GGGCGCCAAAAGCCCTGGCCTGGGGTCAAAAGATCTGGGCTCCAGCCCCAGCCG|TTTATGTGACTATTTGAATTTTTTATTTGTGTTT
                                                        >chr9:25184298-25184628 + E=2e-1
ACAATTTAAAGATTTGATAGAAGCAACCCAAGTTTTTGTTTATTTATTTACCTGTCGATGAATGGATAAAGAAAACGTGGTATATACAT
86
AAAATAAGTTATTAATTGACTTTATAAAGGAAGAAAATTTGGACTCATACCACAATATGAATAAACTTTGTGGACATTATGCTAAATGA

AATAAGTCACAACTAAATGACATTTACTGTAGGATTCACTTGATATGAGGTATCTTGTGTAATCAAACTCATAGAAATAGAAAGCATAA

TGGTAGTTGCCAGGGGCTGGGAGGAAGGA
```
